# Impact of ritual pollution on lactation and breastfeeding practices in rural West Bengal, India

**DOI:** 10.1186/1746-4358-4-2

**Published:** 2009-03-26

**Authors:** Mridula Bandyopadhyay

**Affiliations:** 1 Mother and Child Health Research, La Trobe University, Melbourne, Victoria, Australia

## Abstract

**Background:**

Breastfeeding in India is universal and prolonged. Several cultural practices are associated with lactation and breastfeeding in India, mainly revolving around the concept of ritual purity and 'hot and cold' foods, food avoidance, restricted diet after childbirth, and remaining in seclusion for a certain time period because of the polluting effects of childbirth. This study on breastfeeding practices explored how the concept of ritual pollution influenced practices after delivery, including during lactation and breastfeeding.

**Methods:**

The study was conducted in four villages of West Bengal State in India, representing different levels of socioeconomic development, religion, and caste/tribe from September 1993 to April 1994. One hundred households with one woman respondent from each household were selected from each village. Both qualitative and quantitative methods were employed for data collection. A survey questionnaire was administered to 402 respondents and in-depth interviews were conducted with 30 women in the reproductive age group (13–49 years), and 12 case studies were documented with women belonging to different caste, religious, and tribal groups.

**Results:**

Belief in 'impurity and polluting effects of childbirth' necessitated seclusion and confinement of mothers after childbirth in the study villages. Breastfeeding was universal and prolonged, and food proscriptions were followed by mothers after childbirth to protect the health of their newborn. Initiation of breastfeeding was delayed after birth because of the belief that mother's milk is 'not ready' until two-to-three days postpartum. Generally, colostrum was discarded before putting the infant to the breast in the study villages. Breastfeeding lasted up to five years, and the majority of women in the sample introduced supplementary food before six months. Most infants in the study villages were given a prelacteal feed immediately after birth, only a small number of women (35) exclusively breastfed – after giving a prelacteal feed – until six months in the study villages.

**Conclusion:**

Cultural and traditional practices have considerable implications on lactation and breastfeeding, and in the overall well-being and health of mothers and infants. Breastfeeding programs should take into account traditional beliefs and concepts when communicating with families about practices such as food restriction and food avoidance.

## Background

Breastfeeding is universal in India [1-3] and several cultural practices are associated with lactation and breastfeeding. Cultural practices related to lactation and breastfeeding in India primarily revolve around the concept of ritual purity and 'hot and cold' foods, food avoidance, restricted diet after childbirth, and remaining in seclusion for a period of time due to the polluting effects of childbirth. The concept of purity, defilement and pollution – which is menstruation, birth, and death related – plays a vital role in women's life, particularly after childbirth, thus influencing lactation and breastfeeding.

Cultural restrictions related to food are selectively imposed on women, particularly on widows, pregnant women and women in the initial stages of lactation [4-9]. These practices are upheld and enforced by mothers-in-law, aunts and other elderly female relatives in the family who usually decide the kinds of food a postpartum woman will have after childbirth – which is based on the concept of 'hot and cold' foods. This paper draws on a larger study that examined the socioeconomic and cultural factors influencing maternal and child health care practices. This paper particularly focuses on breastfeeding practices in rural West Bengal and explores how the concept of ritual pollution and 'hot and cold' influences practices after delivery, including during lactation and breastfeeding, which has significant impact on maternal and child health.

### Breastfeeding in India

It is now well established that breastfeeding is universal in India – both in urban and rural areas – and continues into early childhood years; and plays an important role in the context of child health [10-12].

Despite the benefits that breastfeeding provides and its universality, the study of the social aspects of breastfeeding has remained neglected in India. Studies on breastfeeding in India have not taken into consideration the influence of certain cultural and traditional practices on lactation and thereby on breastfeeding.

### Breastfeeding initiation

Initiation of breastfeeding after birth is considerably delayed in India, and in most cases the valuable colostrum is discarded before putting the child to the breast [2,13]. Colostrum is regarded as a yellowish coloured fluid that is harmful to the child's health; hence it is not fed [2]. Whereas, current evidence shows that colostrum contains immunoglobulins, lactoferrin and lysozyme which may help reduce and protect against neonatal septicaemia, diarrhoea, and acute respiratory infections, thus reducing infant mortality rates [14].

Though the advantages of breastfeeding are significant, the duration and patterns of breastfeeding vary a great deal within India. Studies indicate exclusive breastfeeding until four-to-six months of age to be beneficial to infant survival, but globally exclusive breastfeeding rates are still too low in early infancy [15-17].

### Concept of ritual pollution

The concept of ritual pollution is a powerful and enduring social control mechanism in Indian society; concepts of purity, pollution, and defilement are a reflection of cultural attitudes to the body and the organisation of these attitudes into a status hierarchy [18]. Pollution in Indian society can be either temporary or permanent. Specific persons become temporarily impure due to menstruation, birth or death in the family, and contact with lower castes, which makes them temporarily unable to perform certain social and ritual duties. The polluting person either develops some bad condition or simply crosses some line which should not have been crossed and this displacement releases danger for others. Pollution can be committed intentionally, but is more likely to happen unintentionally, and can be neutralised through ritual mechanisms, thus allowing overt action to be taken [18]. For example, temporary pollution and impurity can be removed by performing certain purificatory rituals. But permanent pollution is intrinsic to the caste system, to the division of labour and social control which cannot be altered by purificatory measures. The length of incapacitation because of temporary pollution varies according to a person's caste, occupational class, place of residence and socioeconomic status in the community [19].

Women in India, particularly from disadvantaged and lower socioeconomic strata are often confronted with numerous socio-cultural factors that negatively impinge upon their physical well-being. For a woman in traditional Indian society the childbearing years are the most risky, as they are restricted and governed by a number of taboos relating to their behaviour, movement, decision-making, and status within the family, type of clothes they wear and food they consume, as they are seen as having the power to pollute men. Female sexuality is associated with pollution because of their biology and other circumstances. When associated with pollution, women are perceived as being in danger as well as endangering others [20]. Therefore, women are secluded and confined after childbirth to prevent the spread of polluting effects of afterbirth [6,9,21,22].

Women in traditional Indian society have been conditioned to zealously maintain and observe these taboos, and ensure that the cultural norms, customs and traditions are passed on to the next generation of women. Lawrence argues that women's behaviour can be explained by their conscious choice of modes of behaviour reflecting strategic goals important to their own perceived self-interest [23]. Women are the principal actors in maintaining taboos because it allows them to control certain social interactions outside the household. According to Lawrence, women's behaviour should be viewed as embedded within the complexity of the social relations and values that surround them, rather than as a simple function of ritual pollution. The biological body is commonly seen as a symbol for the social body and bodily control symbolises social control [23].

## Methods

The general aim of the study was to examine the socioeconomic and cultural factors influencing maternal and child health care practices in rural West Bengal. Of the 17 districts in the state, four were chosen on the basis of socioeconomic development, access to health care institutions, transportation and communication, religion, caste, tribe, etc. Accessibility to these areas was also taken into consideration.

A face-to-face survey questionnaire was administered by the researcher (because of low literacy levels amongst the women in the study population) in Bengali. Bengali is commonly spoken in West Bengal, and the researcher is fluent in Bengali. In-depth interviews and case-studies were conducted with women to gain an understanding and insight into various cultural and traditional practices associated with reproductive health, health-seeking behaviour, children's health, including breastfeeding and lactation. Field work for the study was conducted from September 1993 to April 1994. The Ethics Committee of City University of Hong Kong approved the project.

### Survey questionnaire

The questionnaire was pre-tested in a district of West Bengal amongst a small sample of ten women between the ages of 15–45 years to test the efficiency and consistency of the questions, and was amended after piloting. The survey questionnaire was administered to 402 respondents. The survey questionnaire contained 184 questions (structured and open-ended) and took about 90 minutes to complete. The questionnaire was divided into four sections: Section A collected information on socioeconomic and household characteristics of the study sample; Section B was devoted to information on health-seeking behaviour and knowledge and perceptions about diseases and illnesses; Section C elicited information primarily from women respondents on their reproductive health; Section D gathered information on children's health – birth weight, breastfeeding and weaning practices, illnesses during infancy, under five mortality, utilisation of health care services, and immunization status.

### Data collection

One hundred households were randomly selected from each village. From each household, one woman respondent was selected (if two eligible women were part of the same household, the woman who had most recently given birth was included in the study, to avoid recall lapse relating to feeding practices) on the basis of her marital status (currently married), age (between 13 and 49 years), and parity (at least one living child at the time of field research). Consent from participants was audio-taped before administering the face-to-face questionnaire and before conducting in-depth interviews.

Ethnographic tools and techniques (participant observation, interviews, field notes, and case-studies) were employed in gathering data. The in-depth interview guideline was developed by reviewing the humoral medical theory and the concept of hot and cold; concept of ritual pollution; and critically reviewing the framework developed by McCarthy and Maine [24] for analysing the determinants of maternal mortality and morbidity. In-depth interviews were conducted with 30 women (data saturation achieved) in the reproductive age group of 13–49 years on reproductive health; maternal health and nutritional status; child health care practices, including breastfeeding and weaning practices. Twelve case studies were documented with women belonging to different caste, religious, and tribal groups. Both qualitative and quantitative methods were employed for data collection [25].

### Data analyses

Quantitative data were analysed with the Statistical Package for the Social Sciences (SPSS), and was limited to contingency tables and tests of significance (Chi-square). In-depth interviews and case studies were manually analysed by reading and re-reading the transcripts. This enabled identification of emerging analytical categories and also for iteration [26,27], i.e., analytical categories were identified by repetition of a sequence of operations, each building on the one preceding, to achieve results successively. Transcripts were coded and analysed thematically to retain access to the respondent's own categories [27]. As new categories emerged, previously coded data were recoded and re-organised. Themes and sub-themes were cross-checked across the transcripts for consensus. Only major themes are reported in this paper.

For the purposes of the present study exclusive breastfeeding and supplementary feeding are defined as:

• Exclusive breastfeeding: women who only breastfed their infant until six months (including prelacteal feed, because of universality of prelacteal feed)

• Supplementary feed: any feed introduced between one-to-six months besides breast milk.

### Study villages

Detailed description of the study villages are reported elsewhere [25]. (See also Additional File 1). The study was conducted in four villages – Motipur (Tribal), Kapgari (Hindu), Santoshpur (Muslim) and Sultanpur (Mixed).

In this paper, I specifically focus on practices related to seclusion and confinement of the mother and the newborn after birth, dietary behaviour of postpartum women, food avoidance and restriction during lactation, initiation and duration of breastfeeding, and other cultural practices associated with lactation and breastfeeding.

## Results

### Isolation of mother and child after childbirth

The main reason for immediately isolating the mother and the newborn from the rest of the household after childbirth is because of the 'impurity and polluting effects of childbirth', which is believed to be dirty, defiling and contaminating. The following account illustrates the extent of belief in the concept of impurity and pollution after childbirth.

"Someone was sent to fetch the *dai *[traditional birth attendant] during my labour pains, but in the meantime I had given birth. No-one attended to me or attempted to clean me or my baby. I was lying on the mud floor in a pool of blood and placenta with the baby still attached to the umbilical cord. When the *dai *arrived after an hour she cut the cord and cleaned me and my baby."

But some women believed that segregation was practiced to ensure that the mother and the baby were kept away from infections, and that it gave mothers time to recuperate after childbirth.

"Although we do believe in ritual pollution after childbirth, but in our household we practice isolation and segregation of the newborn and the mother primarily to keep them away from infections. As you know, as per our tradition, after birth we have too many visitors coming to 'see the baby'. At this stage, if the infant and the mother are exposed to all the visitors, they would get tired very easily and also may be exposed to certain infections like the cold and the 'flu germs, and as they are vulnerable at this stage it is best to keep them away from interacting with anybody for at least 21 days after birth."

Women in Motipur and Kapgari villages believed in the concept of pollution and contamination after childbirth, which is dirty and defiling (postpartum blood); whilst women in Santoshpur considered the placental blood to be polluting, whereas in Sultanpur women practised segregation as a 'cultural tradition'. Although belief in pollution and impurity after childbirth exists in the study villages, the length of seclusion and the type and place chosen for confinement and amenities and facilities made available to women and the newborn vary significantly from one place to the other and also between communities and caste groups (this has been discussed in detail elsewhere [25]).

### Food avoidance during lactation and breastfeeding

One thing that was common across the study population was the observance of strict food taboos after childbirth. Women in the study villages avoided certain kinds of food, which – according to them – were harmful for the infant. They avoided food believed to have laxative properties, food considered to be cold, food that caused skin rash, and acidic food. (Foods that are generally avoided are: certain varieties of green leafy vegetables, fibrous vegetables, melons, gourds, pumpkin, papaya, eggplant, shell fish, eggs amongst certain caste and communities, certain varieties of fish, lemons, limes, oranges, grapes, chillies, bell peppers, spices, bananas, yoghurt, and oily food).

Women consumed special food (milk, ghee, butter, and certain types of fish) believed to increase the quantity of breast milk and to improve their health. During this period mothers are encouraged and allowed to eat lots of garlic, which is believed to enhance the process of "drying of the womb" (contraction of the uterus). In the study villages, women strictly followed the cultural taboos related to food avoidance and restriction:

"After delivery for the first three days I was on a diet of dry food, such as rice crisps, garlic, and ghee (clarified butter), and was allowed to eat only once a day, as this diet helps to contract the uterus quickly."

In the villages of Motipur and Kapgari mothers were on a diet of rice crisps/puffed rice, tea and hot water for the first three days after childbirth. On the fourth day when the mother completed the purificatory ritual (involving a ritual bath and clipping of nails) she is allowed a mid-day meal of rice and boiled vegetables and lentils (depending on the economic status of the household, meat and fish, may be included). In the night, rice crisps/puffed rice, tea and hot water are allowed until the last day of confinement. Restrictions related to food intake and food avoidance these days – according to the women in Motipur – is dependent on the family's caste and socioeconomic status and standing in the community. Women avoided 'cold' foods to prevent illness after childbirth. Few women from Motipur and Kapgari villages did not follow the practice of food avoidance and diet restriction of eating once a day.

Whilst in Santoshpur village mothers were allowed to eat only those foods considered to be good for her and her child's health. Most women had rice, boiled vegetables, meat, fish, or eggs once a day. In the evenings they had rice crisps/puffed rice, tea, and hot water. This kind of diet continued until the last day of the seclusion period, which is 40 days in the Muslim community. In Sultanpur village the majority of the women ate everything (did not avoid any food) once a day for the entire period of seclusion, and few women had only rice crisps/puffed rice, tea, and hot water for the specified length of confinement and avoided certain foods; this varied between 21 and 30 days. Women from this village who practiced food avoidance and restriction said that they did so because of 'acidity' and 'heartburn'. Women were allowed two meals a day after the completion of their confinement period, but depending on the health of their infant, their diet was constantly altered to protect their infant's health.

Women who had any special food during the initial stages of lactation were from the upper socioeconomic group and women who had delivered at their natal homes. Special foods included were: milk, fruit, various types of pulses and lentils, meat, fish, etc. The women in Sultanpur were provided with an additional supplementary diet from their Anganwadi Centre (Integrated Child Development Scheme – a UNICEF initiative now run by the Ministry of Health and Welfare, Government of India). Women consumed special foods to increase the quantity of breast milk.

### Initiation of breastfeeding

"The first feed my son received was hot water a few hours after birth. I put my son to breast after two days, as the milk 'comes' only after 48 hours. Before I put him to breast I squeezed the yellowish liquid and threw it, as it is harmful for his health because he would not be able to digest this thick yellow liquid".

Breastfeeding initiation was delayed in the study villages. The infants were given prelacteal feeds before breastfeeding. Prelacteal feeds (hot water, sugar-water, honey, mustard oil, tea, or goat/cow milk) were given to the infant to cleanse their system; there was a belief in the study villages that the child swallows waste and impurities in the womb. Only 16.5% initiated breastfeeding within an hour of giving birth (Table 1). About half did not start breastfeeding until at least 24 hours after the birth (47.9%). Women gave a number of reasons for delaying breastfeeding, but the universal reason was that 'it was harmful for the baby' and that there was 'insufficient milk'; colostrum was expressed and discarded before breastfeeding was initiated.

**Table 1:** How long after birth infant was put to breast

**Breastfeeding initiation**	**Motipur** **(n = 98)**		**Kapgari** **(n = 98)**		**Santoshpur** **(n = 92)**		**Sultanpur** **(n = 94)**		**Total** **(n = 382)**	
	**n**	**%**	**n**	**%**	**n**	**%**	**n**	**%**	**n**	**%**
**< 1 hour**	1	1	17	17	21	23	24	26	63	16.5
**1 hour – 24 hours**	41	42	26	27	26	28	43	46	136	35.6
**> 24 hours**	56	57	55	56	45	49	27	29	183	47.9

### Duration of breastfeeding

Breastfeeding was universal, on demand and prolonged – sometimes lasting up to four to five years. The following illustrates this point:

"I breastfed my last born on demand for the first 12 months and thereafter 'til the time she wanted to feed I fed her. I think I breastfed her for about three to four years".

A negligible minority (1.3%) never breastfed (Table 2), and the reasons were: 'insufficient milk'; 'too weak/ill' to breastfeed; and the 'child refused to suckle'. Very few mothers stopped breastfeeding because they were gainfully employed outside home.

**Table 2:** Duration of breastfeeding the last child

**Breastfeeding duration**	**Motipur** **(n = 98)**		**Kapgari** **(n = 98)**		**Santoshpur** **(n = 92)**		**Sultanpur** **(n = 94)**		**Total** **(n = 382)**	
	**n**	**%**	**n**	**%**	**n**	**%**	**n**	**%**	**n**	**%**
< 1 year	0	0	0	0	18	20	7	7	25	6.5
1 year	5	5	7	7	14	15	18	19	44	11.5
2 years	12	12	5	5	0	0	0	0	17	4.4
3 years	12	12	6	6	0	0	16	17	34	8.9
4 – 5 years	19	19	19	19	0	0	8	9	46	12.0
Until child died	2	2	1	1	0	0	3	3	6	1.6
Never breastfed	0	0	4	4	1	1	0	0	5	1.3
Continuing to breastfeed	48	49	56	57	59	64	42	45	205	53.7

### Introduction of supplementary foods

Supplementary food was given to a majority of infants within the first six months (Table 3). No gender discrimination was found in the study population with regards to breastfeeding, or in the introduction of supplementary food.

**Table 3:** Supplementary food given besides breast milk within the first six months

**Supplementary feed**	**Motipur** **(n = 98)**		**Kapgari** **(n = 98)**		**Santoshpur** **(n = 92)**		**Sultanpur** **(n = 94)**		**Total** **(n = 382)**	
	**n**	**%**	**n**	**%**	**n**	**%**	**n**	**%**	**n**	**%**
Yes	89	91	89	91	79	86	86	92	343	89.8
No	9	9	9	9	12	13	5	5	35	9.2
Infant died	0	0	0	0	1	1	3	3	4	1.0

"We had an *annaprasan *[receiving food grains, especially rice] ceremony for our son in the ninth month according to our tradition [for daughters this ceremony is held in the sixth month]. As per our custom, my brother fed the first morsel of rice, fish and sweets to my son. Before the *annaprasan *I was not allowed to give solid food (rice, fish, and vegetables) to my son. Besides breastfeeding, I normally gave him water, animal milk, and fruit juice.

The discrepancy in holding the *annaprasan *ceremony is because of the belief and view of the study sample that male infants are 'weaker' than female infants, and also that male infants are more susceptible to die before completing the first six months of their lives. If they survive past the six months period, then the *annaprasan *ceremony is observed. Some communities observe this in the seventh month and some in the ninth month.

In all the study villages with the exception of Sultanpur, babies were first introduced to supplementary food between the fourth and the sixth months and were given plain water, animal milk, and infant formula. The women in Sultanpur introduced supplementary food for the first time between the first and the third month in the form of plain water, animal milk, and infant formula. Most women in the sample introduced soft mushy foods between the ages of seven and ten months.

Introduction of early supplementary foods for the present study was classified as anytime between one and six months. Exclusive breastfeeding included prelacteal feeds as prelacteal feeds were almost universal in the study population. Only three women in the study sample did not give any prelacteal feed to their newborn; and 35 women exclusively breastfed their babies until six months.

Termination of breastfeeding amongst the study sample was mostly due to insufficient milk, conception and refusal of the child to be breastfed.

## Discussion

Women act upon their household's and traditional beliefs regarding both physical and metaphysical changes that occur during pregnancy and delivery, and believe in ritual pollution and vulnerability during pregnancy and after childbirth [9] thus necessitating seclusion and confinement after childbirth. Generally, women and their newborn are secluded from the rest of the household to limit contamination from the polluting powers of 'after-birth'. This is widely practiced across South Asia, and is an intrinsic part of women's daily lives in traditional societies [4,19,20,22,23,25,28]. However, the interpretation of pollution after childbirth differs significantly, for example, the Hindu women consider the postpartum blood to be polluting and contaminating; whereas the Muslim women in India consider the 'placental blood' to be polluting and contaminating. Interestingly, with increasing socio-economic standing women's perception of 'ritual pollution' changes and they practice this ritual strictly as a 'cultural tradition' and norm rather than believing in the concept of pollution. Nevertheless, the practice of segregation and confinement after birth ensures that the postpartum women get enough rest and avoid contact with carriers of infection [9]; and this ultimately helps them in bonding with their infants.

Dietary restrictions and precautions are the norm in Indian society and are maintained to ensure a healthy pregnancy, safe delivery and quick recovery from childbirth [25]. Persons affected by either birth or death in India avoid certain foods considered to be harmful and are on a restricted diet [19,20,22] based on the concept of 'hot and cold'. Hence dietary restrictions and precautions are observed post childbirth in India to: "dry the womb", recuperate and initiate lactation, and, more importantly, avoiding certain foods to protect the health of the newborn. Food avoidance at the time of lactation is primarily perceived to be in the best interest of the baby, to whom harmful influences could be transmitted through the breast milk [6].

The daily diet of rural women is seldom nutritionally adequate; it becomes less so during pregnancy and lactation. Postpartum mothers in the rural areas of India are not allowed a substantial meal for the first few days after birth; this practice however varies across the country and nowadays depends on the socioeconomic status, caste and religious affiliation. For example, the upper caste Hindu women remain in seclusion and restricted diet for about 21 to 30 days compared to 9 to 11 days for women from the lower castes. Muslim women have a longer period of restricted diet and seclusion (40 days) after childbirth. Contrary to cultural beliefs, dietary restrictions deprive postpartum and lactating women of some of the more nutritious ingredients at a time when they are particularly needed. The quality and quantity of breast milk is also possibly adversely affected by such a diet.

Breastfeeding initiation is delayed across the country because of the belief that mother's milk does not "come" at the time of childbirth; but flows two to three days later [6]; and as the yellowish thick liquid is harmful and difficult for the baby to digest, this is discarded. Prelacteal feeds could be potentially harmful to the newborn as they could introduce infection, sensitise the gut to foreign proteins, or delay the onset of lactation [13]. Delay in initiating breastfeeding may also affect the quantity of breast milk produced because of the delay in stimulation normally provided by suckling [29], and could lead to hypoglycaemia, hypothermia, and acidosis, especially among high risk, low birth weight infants [14,30].

Breastfeeding is extensive in India, however, the exclusive breastfeeding rate was 19% in 1998–1999 at six months [31], and in 2005–2006 it was 46.3% at less than six months [32]. The majority of mothers in my study sample had introduced early supplementary feeds because of the belief that breast milk did not provide adequate nutrition and sustenance to their infants. Supplementary food is generally given after cooking, and usually there is no fixed time for feeding the infants, it often coincides with the food timings of the adults or when the baby cries. Intermittent feeds mainly consist of plain water, infant formula, other liquids, animal milk, and soft mushy foods [33].

Termination of breastfeeding in developing countries is mostly due to perceived insufficient milk, conception and refusal of the child to be fed [33], and this was the case in the present study. Although a well-nourished pregnant woman can successfully continue to breastfeed, however, for women belonging to disadvantaged socio-economic backgrounds and residing in rural areas or urban slums of India, this can be potentially harmful to their already compromised health status. Poor and under-nutrition for women in disadvantaged settings in India does not necessarily begin in the child-bearing years, but commences almost from their birth – with differential treatment in food intake leading to malnutrition, under-nutrition, including anaemia; and differential access to health care. Additionally, consequences of early age at marriage and early pregnancies, short birth intervals, and repeated childbearing to produce a male child (because of 'son-preference') all contribute to rapid maternal nutritional depletion. The practice of 'eating down', i.e., eating less during pregnancy; practices relating to food proscriptions and food avoidance, status of women in the household and society at large, physical workload, and other social, economic and cultural discrimination all pose high risks to the health and well-being of women and her ability to breastfeed during pregnancy without hampering her own health [25,34-36].

One important limitation of this study is that it presents only the household's and women's views relating to the local belief and cultural practices followed after childbirth. Views of health care providers (particularly postnatal nursing staff and midwives) were not collected, which could have provided greater understanding of the social and cultural context in maternal and child health care practices postpartum.

## Conclusion

Undoubtedly breastfeeding is invaluable in the developing world, particularly amongst the lower socioeconomic and disadvantaged groups. But the cultural practice of food avoidance of many nutritious foods and restrictive diet could affect the overall health and well-being of both the mother and her infant. The practice of withholding the breast after birth, discarding valuable colostrum, and giving prelacteal feeds to the newborn needs to be urgently addressed through programs and breastfeeding interventions that infiltrate to the rural areas and urban slums across the country. Messages relating to food restrictions and avoidance should be another intervention health care workers take into consideration to improve the overall health of postpartum mothers and their infants.

The practice of introducing early supplementary food is another major concern in terms of infant health. Messages and importance of exclusive breastfeeding until six months is probably not reaching the grassroots level as women in the present study felt that breast milk did not provide adequate nutrition and sustenance. Breastfeeding promotion and intervention activities in India should take into account the cultural and traditional practices that impact on postpartum women's health and the belief that breast milk is insufficient for an infant until six months.

## Competing interests

The author declares that they have no competing interests.

## Supplementary Material

Additional file 1 **Thumbnail sketches of the study villages**. Click here for file
