# Supplementary material for: Impact of ritual pollution on lactation and breastfeeding practices in rural West Bengal, India
Source: Int Breastfeed J. 2009 Mar 26;4:2. doi: 10.1186/1746-4358-4-2 (PMC2667394; doi:10.1186/1746-4358-4-2)
Supplement: Additional file 1 — Thumbnail sketches of the study villages. [file 1746-4358-4-2-S1.doc]

### Thumbnail sketches of the study villages

A succinct description of the study villages is presented here (for more detailed description see [1]). All the study villages had a primary health centre (PHC), animal husbandry and veterinary hospital, cooperative stores, block development office, ‘*gram panchayat office*’ (elected local self-government), post office, revenue office, ration shop (where essential supplies and food can be purchased at government controlled rates), and a state bank (where agricultural and other loans can be obtained). Other facilities included a land reform office (state government policy is to redistribute land to the landless, agricultural labourers, and to economically and other ‘disadvantaged’ classes); and primary, secondary, and higher secondary schools; and colleges (all within a distance of 5 km or less).

The noticeable differences in the study villages are briefly described, particularly differences in accessibility and availability of communication and transportation.

*Village Motipur, District Puruliya,* is about 220 km west of Calcutta. It is predominantly an agricultural district, but irrigation facilities are almost non-existent. Motipur is about 34 km from the nearest town and is not well connected by road or railway. The village is 5 km from the block headquarter, where many modern facilities are located. The village is poor (all the huts are made of mud and are thatched); the compounds/yards of the houses are kept clean by constant sweeping and coating of the courtyard with cow dung and water. The huts are neat and clean, but dark inside. The village is almost entirely inhabited by scheduled tribe and scheduled caste population (scheduled castes and [scheduled tribes](http://en.wikipedia.org/wiki/Scheduled_tribes) are [Indian](http://en.wikipedia.org/wiki/India) communities that are accorded special status by the [Constitution of India](http://en.wikipedia.org/wiki/Constitution_of_India)). Strangers are looked upon with suspicion in the village, and are not welcomed. Both men and women are reluctant to talk to strangers (this directly relates to their experiences during the ‘Emergency Period’ in India, which began in June 1975 and lasted for about 19 months, when many villagers were forcibly sterilised, see [2]). The village is very detached from the outside world.

*Village Kapgari, District Medinipur,* is about 184 km far southwest of Calcutta. The village is about 24 km from the sub-divisional town, about 8 km from the nearest railway station, and 2 km off the national highway. The bus service into the village, four times a day, brings in children and youth from neighbouring villages to attend school and college in Kapgari. On the main road of the village there are tiny little shops and tea stalls, a chemist, a bicycle repair shop, and other grocery and stationery stores. The majority of the houses in the village are made of earth with thatched roofs and mud walls and floors. All amenities are located close to the main road. Kapgari also has a kindergarten; a college of arts, science, and commerce; Krishi Vigyan Kendra (Agricultural Research Centre), Seva Bharati (Social Welfare Office), and a youth club (Kapgari Kishore Sangha). Of the households surveyed more than three quarters were Hindus.

*Village Santoshpur, District Murshidabad,* is 232 km north of Calcutta. Santoshpur is about 16 km from the nearest town and is well connected with a network of national and state highways, railways, and bus services to neighbouring towns. The road leading into the village is 18 km from the state highway. The district is prone to sudden flash floods during the monsoons. Agriculture plays a vital role in the economy of the district. Sericulture industry (silk worm breeding and the production of raw silk) is the principal agro-based rural industry, and ivory carving is another important cottage industry in the district. The village is mainly residential, and all the activity and noise are concentrated on the main road. Most of the houses have earth flooring and walls and more than half have thatched roofs. The marketplace and the railway station alongside the village resemble a small satellite town. The market has small stationery stores, groceries, chemists, a homeopathic dispensary, several allopathic clinics, cycle repair shops, snack bars, cloth merchants, a hardware store, and a video parlour. Murshidabad is a predominantly Muslim district and the surveyed population was largely Muslim.

*Village Sultanpur, District Barddhaman,* is approximately 178 km northwest of Calcutta. Sultanpur is about 2 km from the national and state highway, and 18 km from the district city headquarter. The village is well connected by bus routes and railways. The railway station is about 3 km from the village. Both the district headquarter and the sub-divisional headquarter are important industrial towns and railway junctions. Barddhaman is known as the ‘granary of West Bengal’ and has a very good network of irrigation facilities. It is also a major industrial centre in the region and one of the leading coal mining regions in the state. The village is about 2 km from the national highway connecting Delhi and Calcutta. The railway station Memari, is across from the national highway. More than three-quarters of the houses have tiled or concrete sheet roofs, more than half have concrete walls, and one third have concrete flooring and electricity. There are no shops within the village. The centre of activity is the railway station, which is where the cinemas, marketplace, grocers and all other shops, chemists and private doctors, both Western and traditional, are situated. The village has a mix of different religious and scheduled caste and scheduled tribe communities.

# References

1. Bandyopadhyay M, MacPherson S: **Women and Health: Tradition and Culture in Rural India**. Aldershot, UK: Ashgate Publishing Ltd.; 1998.

2. Connelly M: **Population control in India: Prologue to the emergency period**. *Population and Development Review* 2006, **32**(4):629-667.
